# Population Monitoring, Egg Parasitoids, and Genetic Structure of the Invasive Litchi Stink Bug, *Tessaratoma papillosa* in Taiwan

**DOI:** 10.3390/insects11100690

**Published:** 2020-10-12

**Authors:** Yi-Hui Wu, Matthew T. Kamiyama, Chuan-Cheng Chung, Hsy-Yu Tzeng, Chia-Hung Hsieh, Chin-Cheng Scotty Yang

**Affiliations:** 1 Miaoli District Agricultural Research and Extension Station, Council of Agriculture, Executive Yuan, Miaoli 36346, Taiwan; yhw@mdais.gov.tw (Y.-H.W.); thomas00250@gmail.com (C.-C.C.); 2 Department of Forestry, National Chung Hsing University, Taichung 402204, Taiwan; erecta@nchu.edu.tw; 3 Laboratory of Insect Ecology, Graduate School of Agriculture, Kyoto University, Kyoto 606-8502, Japan; matthew.kamiyama.67a@st.kyoto-u.ac.jp; 4 Research Institute for Sustainable Humanosphere, Kyoto University, Kyoto 611-0011, Japan; 5 Department of Forestry and Nature Conservation, Chinese Culture University, Taipei 11114, Taiwan; 6 Department of Entomology, Virginia Polytechnic Institute and State University, Blacksburg, VA 24061, USA; 7 Department of Entomology, National Chung Hsing University, Taichung 402204, Taiwan

**Keywords:** integrated pest management, invasive species, mitochondrial DNA, natural enemies, population genetics, population monitoring

## Abstract

**Simple Summary:**

The litchi stink bug (LSB) was inadvertently introduced to Taiwan recently and has since become a severe pest with substantial economic losses. The aim of this study is therefore to improve our knowledge of this invasive pest through multiple approaches including population monitoring, surveillance of natural enemies, and population genetic analysis. Major findings include: (1) a population fluctuation trend that is largely similar to most native LSB populations, (2) a total of seven egg parasitoid species were discovered, two of which (*Anastatus* *dexingensis* and *A*. *fulloi*) being most abundant throughout the LSB infestation in Taiwan, and (3) the occurrence of multiple introductions of LSB to Taiwan. All these data represent a preliminary yet necessary step for the design of future integrated pest management strategies and would help mitigate negative impacts of this invasive pest in Taiwan.

**Abstract:**

Here we assessed population dynamics, natural enemy fauna (with emphasis on egg parasitoid), and population genetic structure (based on mitochondrial DNA) of the invasive litchi stink bug (LSB), *Tessaratoma papillosa* in Taiwan. Our major findings include: (1) fluctuations of LSB in numbers of adults, mating pairs, and egg masses over a 2-year period in Taiwan generally resemble those in the native populations; (2) *Anastatus* *dexingensis* and *A. fulloi* are among the most dominant LSB egg parasitoids, with the former consistently outnumbering the latter throughout Taiwan; (3) the presence of two genetically distinct clades suggests LSB in Taiwan most likely derived from multiple invasions. All these data practically improve our understanding of this invasive insect pest, particularly its ecological and genetic characteristics in the introduced area, which represents critical baseline information for the design of future integrated pest management strategies.

## 1. Introduction

**Figure 1:**
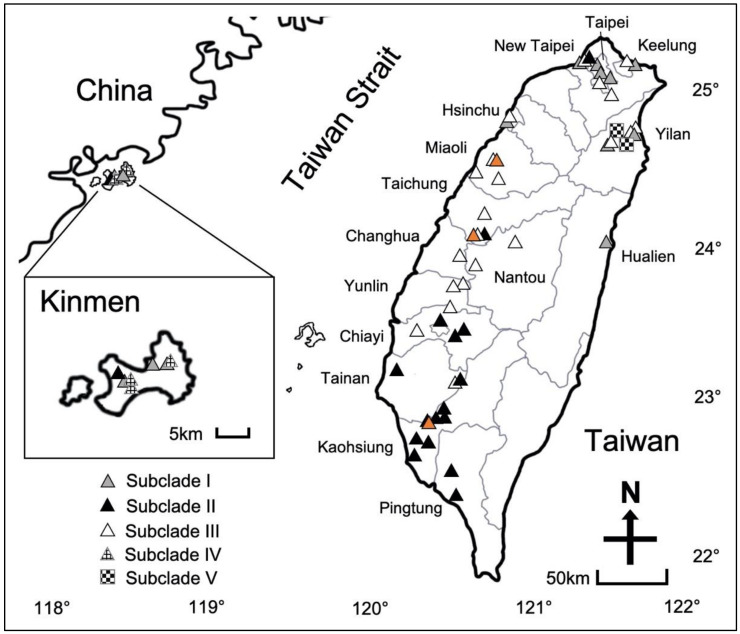
Map showing distributions of collection locality and mitochondrial subclade (I–V) of litchi stink bug (LSB) in both Kinmen and the main island of Taiwan. All county/city names mentioned in the main text are indicated on the map. Orange triangles denote the three sites selected for LSB field monitoring.

The litchi stink bug (*Tessaratoma papillosa* Drury, hereinafter referred to as LSB) is a member of Tessaratomidae that is native to Southeast Asia (e.g., Thailand and Vietnam), India, and several southern provinces of China (e.g., Guangdong, Guangxi, and Fujian) [1,2,3]. In 1997, the LSB was first reported in Kinmen, Taiwan (Figure 1), a continental island that is a few kilometers east of costal line of Fujian Province, China, and was then discovered in the main island of Taiwan (Kaohsiung City, Figure 1) in 2009 [4]. Since its introduction to Taiwan, the LSB has become a severe invasive pest that inflicts substantial economic and agricultural losses. The host plants of the LSB in Taiwan are primarily fruit trees such as litchi (*Litchi chinensis* Sonn) and longan (*Dimocarpus longan* Lour) [5]. Adults or nymphs of LSB have been recorded to damage the shoots, flower spikes, and young fruits of the host plants as their piercing and sucking mouthparts puncture the plant tissues [6], resulting in flower drop, premature fruit drop, wilted twigs and fruits, and black peel. These damages can account for 20–30% reduction in litchi and longan yields, and sometimes can be up to 80% in severely infested areas [7]. The LSB is also considered to be a nuisance pest in urban areas affecting public health. The LSB attacks several native tree species (e.g., flamegold rain tree, *Koelreuteria henryi* Dummer and soap berry, *Sapindus saponaria* Gaertn) that often are used as street trees in numerous urban landscapes such as schools or parks [5]. When disturbed, the LSB releases foul-smelling fluids that are toxic and can irritate the human skin and eyes [8].

The LSB typically possesses a single generation per year, in which post-overwintering adults start being active (feed and mate) and then lay first-generation eggs (F1) in early spring, and F1 adults emerge in late summer/early autumn and overwinter [9]. Studies showed that warm weather conditions appear to trigger earlier adult activity. Most of these data, however, are focused on populations in its native range [9]. Currently, much is unknown about this pest’s population dynamics in newly invaded regions. As pest control measures usually target certain stages in the life cycle of a pest species for a better control efficiency and effectiveness (e.g., egg and pupa parasitoids would specialize host’s egg and pupal stages, respectively), the lack of data involving seasonal population dynamics of LSB in a newly introduced area likely leads to control failure. For example, the crop damage inflicted by another infamous invasive stink bug, brown marmorated stink bug (*Halyomorpha halys* (Stål)), varies with seasonality throughout the year as determined by field experiments performed by Nielson and Hamilton [10]. A better understating of LSB’s phenology in invaded areas is necessary to develop an effective integrated pest management strategy against this invasive pest.

Early control methods against the LSB include chemical (pesticides), biological (natural enemies), cultural (physically removing egg masses) approaches, and in many circumstances combinations of these. While the chemical approach is usually effective in suppressing the local LSB population [11,12], honeybee poisoning as non-target effect during the flowering period of litchi or longan is one unenviable negative consequence [13]. Hence, biological control using natural enemies may play a key role in sustainably reducing the field population of LSB and meanwhile mitigating the effect of chemical control. Previous studies have identified numerous LSB predatory enemies such as: *Tenodera sinensis* Saussure (Mantidae; Mantodea), *Hierodula patellifera* Serville (Mantidae; Mantodea), *Gampsocleis* sp. (Tettigoniidae; Orthoptera), spiders (Araneae), tree toads (Bufonidae; Anura), ants (Formicidae; Hymenoptera) and various bird species [5,9]. Several egg parasitoids of the genus *Anastatus* and *Ooencyrtus* have been recently discovered and used as major biological control agents in a number of IPM projects against the LSB in China [2,9,14]. Nevertheless, information (e.g., fauna, diversity, or phenology) regarding potential natural enemies outside LSB’s native range remains scarce.

While biological control has proved successful in suppressing pest populations in many cases, one challenge remains: the efficacy and success of natural enemies in controlling their host likely depends on an interaction between natural enemy and host genotypes, especially if strong genetic differentiation exists among native populations of an introduced species [15]. It is likely that a widespread enemy species is locally adapted to the host in the native range [16,17]. In such cases, efforts to pinpoint the source population of an invasive pest are of critical importance in identifying appropriate agents of biological control (i.e., strain or genotype that is locally adapted to the host) [16,17,18]. In addition to historical records, marker-based phylogenetic analyses represent the most promising approach to assist in identifying source population and reconstructing introduction pathways of invasive species [17,18]. Considering the broad geographic distribution in its native range, identifying the source population of LSB in Taiwan would facilitate the searching of appropriate strains or genotypes of targeted natural enemies.

The main objectives of this study therefore include: (1) systematic monitoring of the occurrence and determining the population dynamics of *T*. *papillosa* in Taiwan (e.g., number of adults, number of mating pairs and number of egg masses), (2) surveying and identifying egg parasitoids throughout the distribution zones of LSB in Taiwan (both the main island and Kinmen), and (3) assessing the population genetic structure of *T*. *papillosa* based on mitochondrial DNA, with an attempt to identify its putative source population. Data generated in the present study are expected to provide baseline information to support the development and implementation of future control measures against LSB.

## 2. Materials and Methods

### 2.1. Monitoring of LSB Populations

We selected a total of three field monitoring sites in one of three counties (Miaoli, Taichung, and Kaohsiung, Figure 1). The GPS coordinates were 24°33′18″ N 120°45′22″ E, 24°04′02″ N 120°42′43″ E, and 22°51′11″ N 120°20′20″ E for Miaoli, Taichung, and Kaohsiung, respectively. Only the Kaohsiung site is located in tropical climate zone, while the other two sites are considered subtropical. At each site, longan trees were the dominant type of vegetation, which minimizes the effect of different host plant species on the population dynamics of *T*. *papillosa*, if any. Monitoring was performed from January 2018 to December 2019. Monitoring was conducted twice per month (10–14 days apart) from February to June and once per month from July to the following January. In every monitoring event, we counted the number of adult LSB, number of LSB mating pairs, and number of LSB egg masses from up to four randomly selected branches similar in both size and length (with the presence of LSB, if possible). The same procedure was replicated for a total of fifteen longan trees at each site. Each metric (number of adults, number of mating pairs, and number of egg masses) was totaled per tree then averaged across the fifteen trees at each site for the two years and plotted. A two-way ANOVA was performed to determine the effect that year and site had on the number of LSB adults, mating pairs, and egg masses. All the samples were randomly selected and independent to each other. Additionally, residual vs. fitted plots were generated from the data which indicated constant variance was maintained during the statistical analysis. If a significant difference between the measured traits was found (*p* < 0.05), a Tukey’s HSD test was performed to separate the differences between the means.

### 2.2. Survey for Egg Parasitoids

We collected egg masses of *T*. *papillosa* in Taiwan from April to June in 2018 and March to May 2019, respectively. In brief, we selected two sites with longan trees as main vegetation from each of 12 counties/cities across the main island of Taiwan and Kinmen, and a 1-h visual inspection of egg masses was performed within each site twice per month, resulting into a total of 4-h inspection per month in each county/city. Collected egg masses were transferred to the lab and maintained in 9-cm disposable petri dishes at 25 °C until LSB nymphs or adult parasitoids emerged. Once emerged, parasitoid species identity was confirmed by a professional taxonomist, Dr. Gary A.P. Gibson at the Agriculture and Agri-Food Canada, following the species key reported in [19]. We calculated the parasitoid impact [20] by each egg parasitoid species by dividing the number of parasitized eggs over the total number of field-collected eggs. The percentages of hatched and unhatched eggs were also calculated. Two more indices, namely discovery efficiency and exploitation efficiency [20], were employed to evaluate the overall efficacy of each egg parasitoid. The discovery efficiency was expressed by the number of egg masses discovered by the parasitoid over the total number of egg masses, whereas the exploitation efficiency refers to the number of parasitized eggs over the total number of eggs within the discovered egg masses [20]. Note that we only presented data on two major species, *Anastatus fulloi* Sheng and Wang, 1997 and *A*. *dexingensis* Sheng and Wang, 1997, as these two are the most abundant in the field (refer to Section 3.2 for more details). To understand the geographic distribution of egg parasitoids in Taiwan, we pooled the data based on region in which egg masses were collected and averaged. Data derived from egg masses collected from northern Taiwan (Miaoli, Hsinchu, Taipei, and Yilan County/City), central Taiwan (Yunlin, Changhua, Nantou, and Taichung County/City), southern Taiwan (Pingtung, Kaohsiung, Tainan, and Chiayi County/City) and the remote island (Kinmen) were respectively combined. Distances between sites within regions were at least 3–5 km.

### 2.3. Genetic Structure of LSB Based on Mitochondrial DNA

#### 2.3.1. Specimen Collection and DNA Extraction

A total of 81 adult *T*. *papillosa* were collected (n = 69 from the main island of Taiwan; n = 8 from Kinmen), Thailand (n = 2), and China (n = 2) (Table S1, Figure 1). Genomic DNA was extracted from tissue of a single leg of each specimen using Tissue Genomic DNA Extraction Mini Kit (Abundance Life Science, Kaohsiung, Taiwan).

#### 2.3.2. PCR Amplification and DNA Sequencing

The partial mitochondrial cytochrome *c* oxidase subunit I (*COI*) was amplified by PCR with the arthropod-universal primer set, namely LCO1490 (5′-GGTCAACAAATCATAAAGATATTGG-3′) and HCO2198 (5′-TAAACTTCAGGGTGACCAAAAAATCA-3′) [21]. PCR amplifications were conducted using 1 µL of each template DNA in a total reaction volume of 25 µL containing 0.15 µM of dNTPs, 2.5 mM of MgCl, 0.75 units of Taq DNA polymerase, and 0.6 µM of each primer in a Veriti thermal cycler (Applied Biosystems, Foster City, CA, USA). The PCR program was as follows: 1 cycle of 5 min at 95 °C; 40 cycles of 30 s at 95 °C, 30 s at 52 °C, and 45 s at 72 °C; and a final cycle of 10 min at 72 °C. PCR products were purified using a PCR Clean Up System (Viogene, Taipei, Taiwan) and sequenced on an ABI 3730 DNA Analyzer (Applied Biosystems, Foster City, CA, USA) using an ABI PRISM Terminator Cycle Sequencing Ready Reaction Kit, ver. 3.1 (Applied Biosystems, Foster City, CA, USA), and sequencing reactions were carried out by the Genomics Company, New Taipei City, Taiwan.

#### 2.3.3. Sequence Analyses and Phylogenetic Reconstruction

Sequences were assembled using Seqman II software (Lasergene, Madison, WI, USA). A closely related stink bug species, *Tessaratoma javanica* (Thunberg) (KF534917) was selected as an outgroup in the phylogenetic analysis. Sequence alignments were constructed using the default settings in Muscle in MEGA (version 7.0) [22]. The best nucleotide substitution model (the Tamura 3-parameter plus gamma model, T92+G) for phylogenetic analysis was estimated using the ModelTest in MEGA (version 7.0) [22]. Phylogenetic trees were reconstructed using maximum likelihood (ML) with 1000 bootstrap replications for nodal supports in MEGA (version 7.0). Bayesian inference was also used to reconstruct the phylogenetic tree using MrBayes ver. 3.2 [23]. Two runs of four independent Metropolis-coupled Markov chain Monte Carlo (MCMC) analyses were run for 1 × 10^6^ generations and sampled every 1000 generations with a burn-in length of the initial 10% generations. Haplotypes of *COI* of *T*. *papillosa* were detected using DAMBE version 7 [24]. Relationships among haplotypes were inferred by a haplotype network analyses using Network version 10 (https://www.fluxus-engineering.com/).

## 3. Results

### 3.1. Population Dynamics of LSB

**Figure 2:**
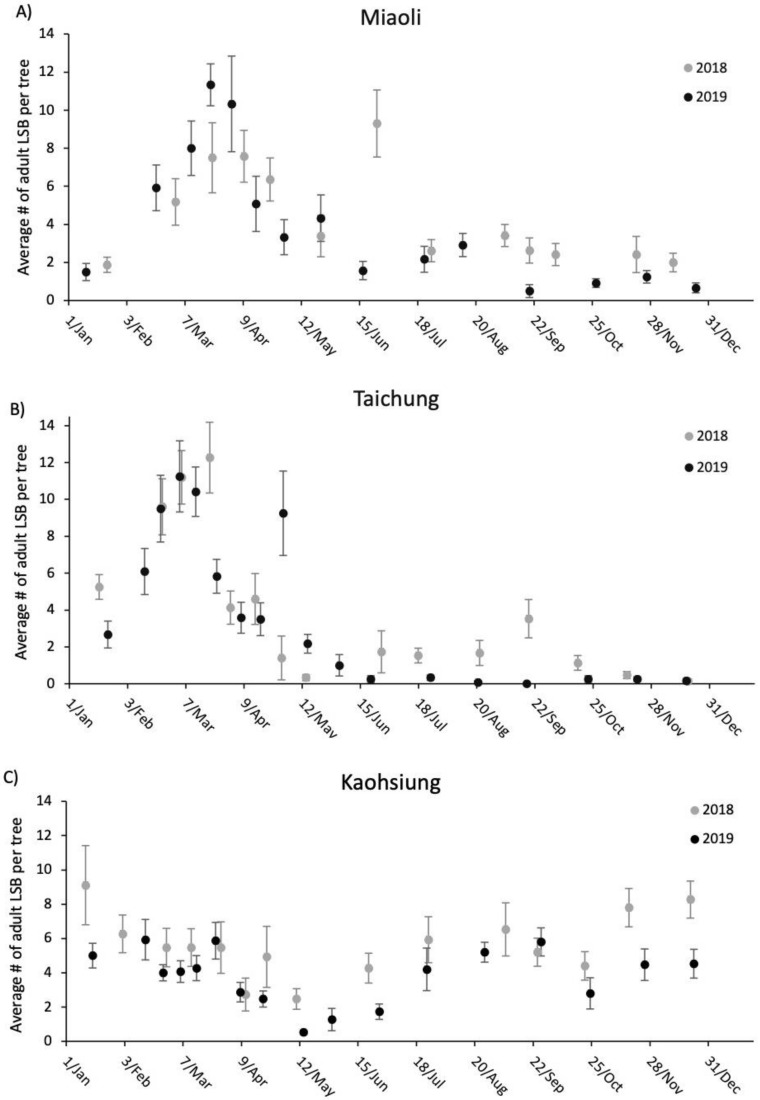
Dot plots with standard error bars of average number of observed adult LSB per tree from January through December at Miaoli (**A**), Taichung (**B**), and Kaohsiung (**C**), for 2018 (grey) and 2019 (black).

**Figure 3:**
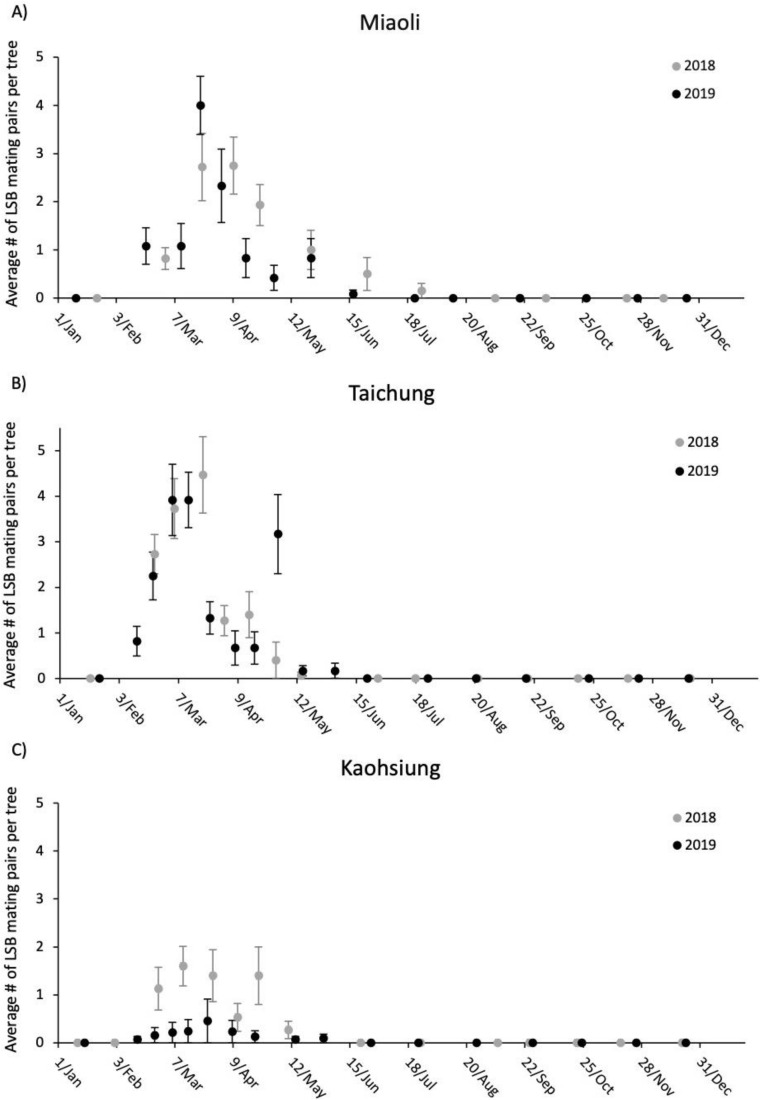
Dot plots with standard error bars of average number of observed LSB mating pairs per tree from January through December at Miaoli (**A**), Taichung (**B**), and Kaohsiung (**C**) for 2018 (grey) and 2019 (black).

**Figure 4:**
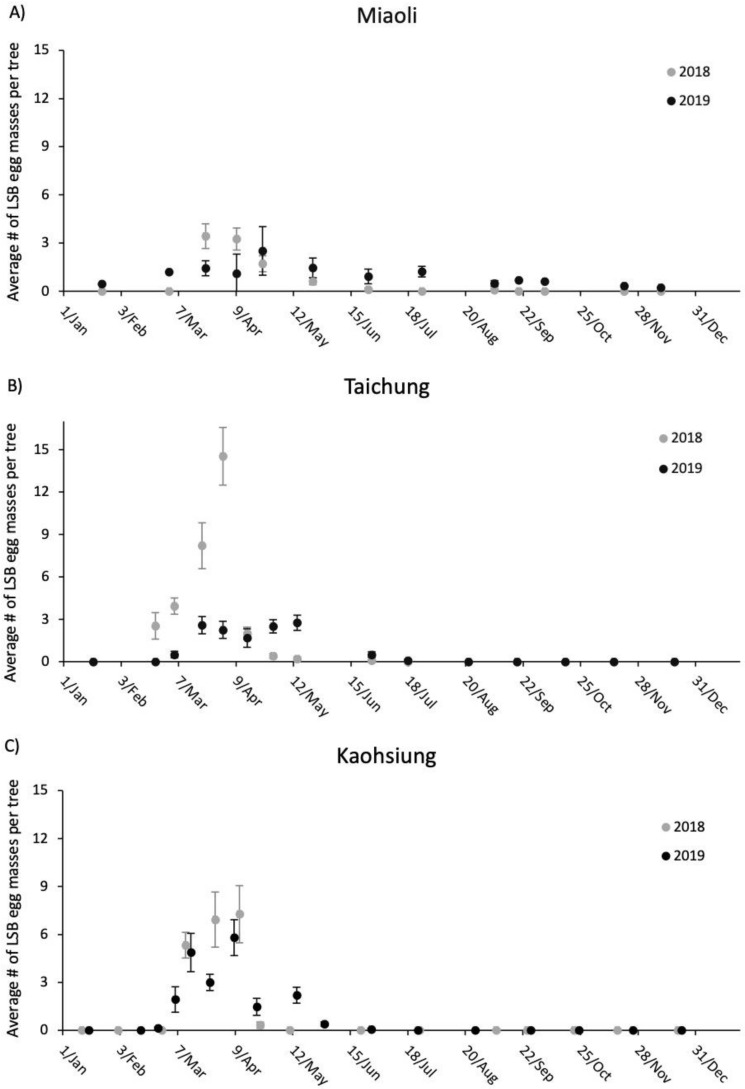
Dot plots with standard error bars of average number of observed LSB egg masses per tree from January through December at Miaoli (**A**), Taichung (**B**), and Kaohsiung (**C**) for 2018 (grey) and 2019 (black).

The adult LSB population fluctuation in both Miaoli and Taichung from January 2018 to December 2019 resembled each other, but LSB from Kaohsiung followed a different population trend (Figure 2A–C). In the former two sites, an increase in adult numbers was observed beginning in January and peaking in March for both years, followed by a decrease in May with a low number of adults in autumn and winter (Figure 2A,B). However, there was no apparent peak in the number of adults at the Kaohsiung site, with the lowest number of adults observed in the early summer. Unlike in Miaoli and Taichung, adult *T*. *papillosa* were regularly spotted during autumn and winter (Figure 2C). Figure 3 showed fluctuations of mating pair numbers and that the three sites possessed a similar tendency in which overwintered *T*. *papillosa* started to mate in February and reached the mating peak in March for both 2018 and 2019. Very few mating pairs were recorded in May, followed by an 8-month (June to the following January) period with no observed mating activities. We began to observe egg masses, albeit low numbers, in February at Taichung and Kaohsiung, but not from Miaoli, where the first egg mass was found in March. March and April were the peak months for the number of egg masses, followed by a rapid decrease in May (Figure 4). In general, the number of egg masses reached the peak roughly two weeks to one month after the mating peak (Figure 4).

Year (2018, 2019) had a significant effect on number of adult LSB observed at the three sites (*p* = 0.042). Site (Miaoli, Taichung, and Kaohsiung) also had an effect, as there was a significant difference in number of adult LSB recorded between Taichung and Kaohsiung (*p* = 0.031). Year did not have an effect on observed number of mating pairs of LSB, however, there was a significant difference in number of observed mating pairs between Miaoli and Kaohsiung (*p* < 0.001), and Taichung and Kaohsiung (*p* < 0.001). Year had a significant effect on number of LSB egg masses observed at the three sites (*p* = 0.011). Site did not have an effect on number of LSB egg masses between years.

Additional observations showed that for both years from February to May feeding and mating by overwintered adult *T*. *papillosa* occurred primarily on young shoots or flowers, whereas from August to January most adults were newly emerged (with a thick, whitish waxy layer under lower surface of the body) and tended to rest on the back of leaves.

### 3.2. Diversity and Abundance of Egg Parasitoids

Of all parasitized eggs collected in 2018 and 2019, we have identified six egg parasitoid species in Taiwan including four monoparasitic wasps, *Anastatus fulloi*, *A*. *dexingensis*, *A*. *formosanus* Crawford, 1913 and *Anastatus* sp. and three polyparasitic wasps, *Ooencyrtus utetheisae* (Risbec), *O. phongi* Trjapitzin, Myartseva, and Kostjukov 1977, and a species belonging to *Eulophidae* (hereinafter referred to as eulophid wasp X, Table S2). Preliminary data suggest that *Anastatus* sp. may contain cryptic species (two putative species may exist; however, evidence accumulation is presently ongoing. In the current study, we treated *Anastatus* sp. as one species), while eulophid wasp X has been confirmed as a single species only (Gibson et al., unpublished data). As the majority of parasitism records belonged to *A*. *dexingensis* and *A*. *fulloi* (>97% of incidences), we only showed the data based on the two species (Table 1 and Table 2). The two species were found to be distributed throughout the main island of Taiwan, with *A*. *dexingensis* consistently outnumbering *A*. *fulloi* during our survey, except Kinmen where only *A*. *fulloi* was found. A higher level of parasitoid impact was generally found in May and June in 2018 but not in 2019 in which the parasitoid impact was low across all regions and months. Although the discovery efficiency of the two egg parasitoids species varied across regions, months and years, two major trends could be observed: (1) the discovery efficiencies of *A*. *dexingensis* tend to be higher than those of *A*. *fulloi* during our 2-year survey (e.g., maximum discovery efficiencies are 29.4 ± 11.4 and 83.3 ± 5.4 for *A*. *dexingensis* and *A*. *fulloi*, respectively); (2) the discovery efficiencies of *A*. *dexingensis* tend to increase with sampling month in 2018 but not in 2019 (Table 2). The exploitation efficiencies seemed to resemble each other between *A*. *dexingensis* and *A*. *fulloi*, however, we found a rough trend that the exploitation efficiency increased with the discovery efficiency during our survey. Numbers of eggs parasitized by the other five “minor” egg parasitoids were summarized in Table S2. All “minor” egg parasitoids were detected at least in one of the regions in the main island of Taiwan but absent from Kinmen.

### 3.3. Genetic Structure of LSB Based on Mitochondrial DNA

**Figure 5:**
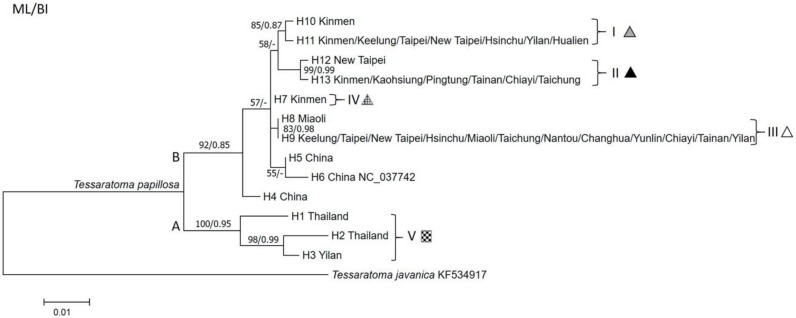
Maximum-likelihood phylogeny of LSB based on mitochondrial *COI* sequences. Numbers at the nodes represent maximum-likelihood bootstrap values and Bayesian posterior probabilities.

**Figure 6:**
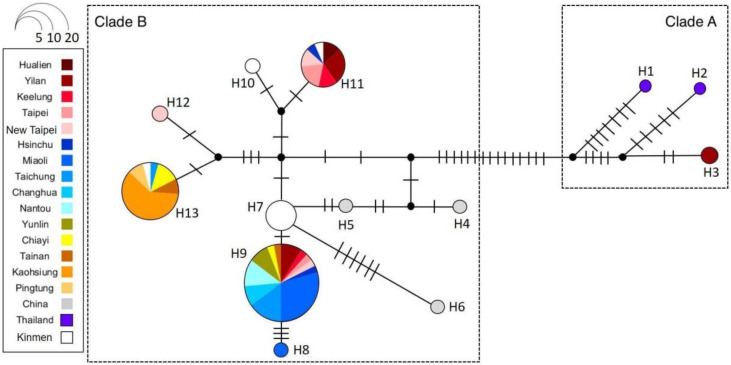
Haplotype network of LSB based on partial mitochondrial *COI* sequences. Each bar on the branch corresponds to a single nucleotide substitution. Circle area is proportional to the number of individuals carrying a given haplotype.

Thirteen *COI* haplotypes were identified from all samples of *T*. *papillosa* in the present study. Phylogenetic analysis based on both ML and Bayesian trees showed that the 13 haplotypes were separated into two major clades (Clade A and B) with high support values (Figure 5). Clade A was comprised of three haplotypes (H1~H3). The haplotypes H1 and H2 were distributed in Thailand, whereas H3 only was distributed in a single collection site in Yilan, Taiwan. Phylogenetic analysis revealed H1 from Thailand was the ancestral haplotype of Clade A (Figure 5). Clade B was comprised of ten haplotypes (H4~H13), with most of individuals from the main island of Taiwan, Kinmen, and China being clustered in Clade B. Branches within Clade B, however, were not well-supported as evident by low bootstrap values and the Bayesian posterior probability values, suggesting a polytomy of Clade B. The haplotype H4 from China was at basal position, thus represented the ancestral haplotype of Clade B (Figure 5). The results of haplotype network analysis (Figure 6) were in a perfect agreement with the ML/Bayesian phylogenetic trees (Figure 5), supporting the occurrence of two major clades of *T*. *papillosa* (Clade A and B).

All *T*. *papillosa* collected from China and Thailand were each represented by a unique haplotype, while samples collected from Taiwan, except Kinmen, were mostly found to share the same haplotypes (e.g., H9, H11, and H13 were recovered from multiple samples in Taiwan, Figure 5). The haplotypes H9, H11, and H13 appeared to be among the most widespread haplotypes in Taiwan. The genetic structure of *T*. *papillosa* in Taiwan could be further divided into five subclades (Subclade I–V, Figure 5) with a rough geographic congruence (Figure 1): (1) individuals belonging to Subclade I were distributed in northern and northeastern Taiwan; (2) most individuals collected from southern Taiwan were grouped in Subclade II; (3) Subclade III comprised individuals collected from western Taiwan; (4) Subclade IV and V each comprised two individuals from Kinmen and Yilan, respectively.

## 4. Discussion

### 4.1. Population Dynamics of LSB

The overall trend of population dynamics of LSB is: (1) mating occurs roughly in mid-February and peaks in March; (2) the peak time for egg-laying is from late March to early April, followed by a significant reduction in early May. This pattern largely resembles that of LSB populations in Hainan, Guangxi and Guangdong Provinces, China [2,25] and Son La Province, Vietnam [26], with the only exception for a delayed mating peak in Guangdong which is most likely the result of this population being located in a higher altitude than all other reported populations. Our statistical analyses indicate that sites generally have significant effect on the three measured metrics except for the number of egg masses, and that virtually all cases of significant pairwise differences involve the Kaohsiung site. Coincidentally, among the three sites only Kaohsiung is located in the tropical region, suggesting the observed differences in trend of populations dynamics between our monitoring sites in Taiwan can be at least partially attributed to factors associated with different climates. All these data (both in the native and invasive range) support the notion that climatic conditions represent key factors to predict population dynamics of LSB and also to develop an appropriate pest management strategy.

Our results on the field monitoring suggest that all metrics in the three sites in Taiwan generally show a similar trend except the number of adult LSB. In the Kaohsiung site, we observed an increase in the number of adults immediately after the major reduction of adult numbers during May for both years, while the number of adults remained low without apparent increase until the following January in Miaoli and Taichung. One explanation is that most F1 adults remain in Kaohsiung after the majority of post-overwintering adults die off, whereas the other two sites may have possessed an environment with favorable conditions for mating, but not laying eggs or overwintering. While empirical demonstration of such dispersal is currently lacking, two lines of evidence may provide indirect support: (1) there were high numbers of adults (post-overwintering adults) and mating pairs in the Miaoli and Taichung sites (Figure 2 and Figure 3), yet the number of egg masses was relatively low (Figure 4); (2) temporal variations in haplotype composition are found in the samples from the same sites collected at different years in both Miaoli and Taichung sites (Figure 5 and Figure 6; Table S1), suggesting that dispersal of new individuals with different genetic make-up may have occurred. Furthermore, a similar dispersal pattern was also observed in the LSB populations in the North Eastern Hill (NEH) region of India [1], suggesting that it is more common than previously thought that egg-laying behavior of LSB may not necessarily occur in the places in which overwintering adults primarily mate. Although data on seasonal migration patterns of LSB are still being accumulated (Wu et al., unpublished data), our study raises the concern for the field release of egg parasitoids that it should be verified whether LSB in a target site tends to stay for “egg-laying” after mating before an egg parasitoid release project is implemented.

### 4.2. Egg Parasitoids as Biocontrol Agents of LSB in Taiwan

Our survey over a period of two years has detected a total of seven egg parasitoid species, two of which, *A. dexingensis* and *A. fulloi*, being the most dominant LSB egg parasitoids. These two *Anastatus* species are of great potential to be integrated into LSB management framework as biocontrol agents. Geographic distribution and at least two parasitoid indices (parasitoid impact and discovery efficiencies) indicate that *A. dexingensis* appears to be more dominant than *A. fulloi* across all collection sites in the main island of Taiwan, while egg parasitoids detected in Kinmen are exclusively *A. fulloi*. The difference in the field abundance of the two egg parasitoid species may provide insightful information concerning the design of an effective, efficient biocontrol project in Taiwan. Competition to monopolize host resources can be predicted when two or more parasitoids of the same or different species attack the same host [27]. Indeed, discovery and exploitation efficiencies for *A. fulloi* are reduced as the two indices for *A. dexingensis* increase. A number of biotic factors have been reported to influence the outcome of such competition between parasitoid species, and these include differences in host searching abilities, reproductive potential, dispersal and fighting abilities, and/or phenological synchronization with the host, along with abiotic factors as well [28,29,30,31]. Future research effort therefore should be focused on empirical tests of multiple factors that potentially shape the outcome of inter-specific competition observed in the field.

Despite being outnumbered by *A. dexingensis* in the field, we argue that the assessment of *A. fulloi* as a potential natural enemy for future field release should not be neglected for several reasons. First, no obvious difference was found in the exploitation efficiencies between *A. dexingensis* and *A. fulloi*, suggesting competence of *A. fulloi* as an efficient egg parasitoid once LSB egg masses are discovered. Furthermore, our phylogenetic analyses reveal that the majority of LSB in the main island of Taiwan are genetically similar to populations in Kinmen (see Section 4.3 for more details). The presence of *A. fulloi* as a sole egg parasitoid species in Kinmen, coupled with a nearly 100% parasitism rate, suggests this species could perform better against the LSB population in the main island of Taiwan due to “genotype matching” between natural enemies and LSB (see Section 4.3 for more details) as a result of co-evolutionary history [32].

### 4.3. Invasion History of LSB in Taiwan and its Biocontrol Implications

Our analyses of the population genetic structure of LSB using mtDNA data indicate the presence of the two genetically differentiated clades in Taiwan, suggesting LSB in Taiwan most likely results from at least two separate invasions. We then inspected the geographic distribution of mtDNA haplotypes which revealed a complex invasion history of LSB in Taiwan. Despite no shared haplotypes, all individuals in Kinmen harbor haplotypes that are most genetically similar to those in China, leading us to conclude that the remote island, Kinmen, appears to receive colonization(s) of LSB from China. Indeed, there have been speculations of China-origin for the LSB in Kinmen [33,34], especially given the close proximity of Kinmen to coastal line of Chinese provinces in which LSB is distributed as well as intensive commerce activities between the island and China. Most LSB individuals in the main island of Taiwan share identical (or similar) haplotypes to those that are present in Kinmen (Figure 1), which itself is an invasive population, suggesting that Kinmen serves as the most likely source population of LSB in the main island of Taiwan. Such introduction pathways in which propagules of exotic species primarily originate from an invasive population rather than from their native ranges is termed “secondary introduction” [35,36]. Increasing evidence has been accumulated to suggest this mode of introduction may be prevailing, and our finding of H9, H11, and H13 being widespread haplotype implies that LSB may have been repeatedly introduced to the main island of Taiwan via “secondary introductions”.

Interestingly, we also found evidence of introductions of LSB from a different part of its native range (e.g., Thailand) into some locations in the main island of Taiwan (e.g., Yilan), raising an immediate concern that gene exchange is allowed to occur between geographically isolated/genetically differentiated populations of LSB and thus genetic diversity can be increased in the “hybrid” zone. While the consequences of population admixture are yet to be determined, several pest outbreak cases have been linked to elevated levels of genetic variation of introduced populations as a result of genetic “hybridization” [37,38]. Empirical data, such as assessment of adaptability or insecticide resistance can be obtained through long term monitoring of populations in northeastern Taiwan (i.e., Yilan, Figure 1) as individuals bearing genetically distinct haplotypes are found in sympatry there.

Our results are highly relevant to the design of control programs against invasive populations of *T. papillosa* using natural enemies. The presence of two major clades with substantial genetic differentiation in invasive populations of *T. papillosa* in Taiwan is of critical importance because success of such enemies in attacking *T. papillosa* may depend on genotype matching between the enemies and LSB if local adaptation to their geographically unique hosts exists [32]. Such “genotype matching” of hosts and parasitoids/parasites has been demonstrated in several systems including phorid flies vs. fire ants [39] and plant pathogens vs. host plants reviewed in [40], and we argue that a sustainable biological control agent is likely to be more effective if they are collected from native range in which specific local variants of *T. papillosa* that were introduced to Taiwan are inhabited (e.g., egg parasitoids from Thailand for LSB population bearing H3 haplotype, Figure 5 and Figure 6).

## 5. Conclusions

This is the first study integrating multiple approaches (population monitoring, survey for natural enemies, and molecular marker-based population genetic analysis) to understand the LSB in its introduced areas, with an ultimate goal of developing a sustaining biologically-based pest management strategy (e.g., release of various natural enemies in conjunction with other methods that are compatible). Future efforts should be directed to test how to maximize the efficiencies of the two major egg parasitoid species based on the knowledge of intrinsic and extrinsic factors that shape their inter-specific competition, to predict the proper timing for release of egg parasitoids taking consideration of on-site population dynamics of LSB, and lastly to determine whether there is “genotype matching” of the LSB and the two major egg parasitoids.

## Figures and Tables

**Table 1:** Percentages of parasitized by each of the two major egg parasitoid species (parasitoid impact), hatched and unhatched eggs in the main island of Taiwan (northern, central, and southern) and Kinmen.

		2018			2019		
		April	May	June	March	April	May
**Northern**							
	No. of eggs	230	753	634	204	1752	1061
	*A.f.*	17.4	5.3	0.2	0.0	0.0	3.7
	*A.d.*	5.7	35.1	58.2	0.0	0.0	2.6
	h	52.6	12.6	2.2	71.1	89.6	83.4
	u	24.3	47.0	39.4	28.9	7.5	10.3
**Central**							
		-	756	14	146	877	908
	*A.f.*	-	3.0	0.0	0.0	5.7	2.9
	*A.d.*	-	48.4	100.0	8.2	29.4	23.1
	h	-	1.7	0.0	89.0	49.8	62.1
	u	-	46.9	0.0	2.8	15.1	11.9
**Southern**							
	No. of eggs	400	140	14	1592	739	426
	*A.f.*	0.0	20.0	0.0	1.9	7.4	3.5
	*A.d.*	11.8	44.3	71.4	1.8	24.2	27.7
	h	67.0	2.1	0.0	75.6	38.7	8.2
	u	21.3	33.6	28.6	20.7	29.7	60.6
**Kinmen**							
	No. of eggs	29	-	-	-	27	-
	*A.f.*	100.0	-	-	-	96.4	-
	*A.d.*	0.0	-	-	-	0.0	-
	h	0.0	-	-	-	0.0	-
	u	0.0	-	-	-	4.0	-

“*A*.*f*.”: *Anastatus fulloi*; “*A*.*d*.”: *A*. *dexingensis*; “hatched”: emergence of LSB nymph; “u”: unhatched (for unknown reasons); “-”: no eggs collected.

**Table 2:** Mean (±SE) discovery efficiency and exploitation efficiency of each of two major egg parasitoids on LSB egg masses collected in this study during 2-year survey (data on the minor egg parasitoid species were excluded from this analysis).

	2018			2019		
	April	May	June	March	April	May
**Northern**						
Discovery efficiency						
*A.f.*	29.4 ± 11.4	16.4 ± 5.0	2.1 ± 2.1	0	0	7.0 ± 2.8
*A.d*.	17.6 ± 9.5	56.4 ± 6.7	83.3 ± 5.4	0	0	8.1 ± 2.7
Exploitation efficiency						
*A.f*.	57.4 ± 13.0	43.9 ± 8.6	7.1 ± 0.0	0	0	47.9 ± 13.0
*A.d*.	38.4 ± 16.5	62.6 ± 5.7	76.1 ± 4.5	0	0	24.1 ± 11.2
**Central**						
Discovery efficiency						
*A.f*.	-	10.7 ± 4.2	0	0	9.0 ± 3.5	6.8 ± 3.0
*A.d*.	-	80.4 ± 5.4	100.0 ± 0.0	9.1 ± 9.1	41.8 ± 6.1	28.8 ± 5.3
Exploitation efficiency						
*A.f*.	-	23.0 ± 12.4	0	0	51.8 ± 12.2	54.8 ± 18.6
*A.d*.	-	59.2 ± 4.9	100	85.7 ± 0.0	69.5 ± 6.4	75.5 ± 6.4
**Southern**						
Discovery efficiency						
*A.f*.	0	27.3 ± 14.1	0	1.7 ± 1.2	17.2 ± 5.0	7.7 ± 4.3
*A.d*.	21.9 ± 7.4	81.8 ± 12.2	100	4.2 ± 1.8	48.3 ± 6.6	43.6 ± 8.0
Exploitation efficiency						
*A.f*.	0	41.9 ± 22.7	0	54.6 ± 22.6	38.1 ± 9.3	43.0 ± 28.8
*A.d*.	63.8 ± 10.9	64.4 ± 12.7	71.4 ± 0.0	41.5 ± 11.2	53.5 ± 5.6	58.7 ± 8.3
**Kinmen** (only *A.f*. was found)						
Discovery efficiency	100	-	-	-	100	-
Exploitation efficiency	100	-	-	-	96.4 ± 0.0	-

“*A.f.*”: *Anastatus fulloi*; “*A.d.*”: *A. dexingensis*.

## Supplementary Materials

The following are available online at https://www.mdpi.com/2075-4450/11/10/690/s1, Table S1: Samples of LSB used in this study and GenBank accession numbers. Table S2. Number of LSB eggs parasitized by “major” (Anastatus fulloi and A. dexingensis) and “minor” egg parasitoid species (A. formosanus, A. sp., Ooencyrtus utetheisae, O. phongi and eulophid wasp X). Note the egg numbers for the “minor” egg parasitoid species (in bold) are not included in the total egg numbers shown in Table 1.
