# Supplementary material for: Population Monitoring, Egg Parasitoids, and Genetic Structure of the Invasive Litchi Stink Bug, Tessaratoma papillosa in Taiwan
Source: Insects. 2020 Oct 12;11(10):690. doi: 10.3390/insects11100690 (PMC7600713; doi:10.3390/insects11100690)
Supplement: Supplementary file 1 [file insects-11-00690-s001.pdf]

Table S1. Samples of LSB used in this study and GenBank accession numbers

No	Sample code	Date	Location	Haplotype	Sub-clade	GenBank accession No.
1	TP01	2018.Apr.16	Kinmen, Taiwan	H7	IV	MT946201-04
2	TP02	2019.Feb.21	Miaoli, Taiwan	H8	III	MT946205-06
3	TP02-1	2020.Feb.29	Miaoli, Taiwan	H9	III	MT946207-08
4	TP03	2019.Mar.30	Dongshan, Yilan, Taiwan	H11	I	MT946209-10
5	TP03-1	2020.Mar.27	Dongshan, Yilan, Taiwan	H9	III	MT946211-13
6	TP03-2	2020.Mar.27	Dongshan, Yilan, Taiwan	H3	V	MT946214-15
7	TP04	2019.Mar.28	Mingjian, Nantou, Taiwan	H9	III	MT946216
8	TP05	2019.Mar.29	Shetou, Changhua, Taiwan	H9	III	MT946217
9	TP06	2019.Apr.03	Xihu, Miaoli, Taiwan	H9	III	MT946218
10	TP07	2019.Apr.09	Tianliao, Kaohsiung, Taiwan	H13	II	MT946219
11	TP08	2019.Apr.10	Beitun, Taichung, Taiwan	H9	III	MT946220
12	TP09	2019.Mar.28	Mingjian, Nantou, Taiwan	H9	III	MT946221
13	TP10	2019.Mar.29	Shetou, Changhua, Taiwan	H9	III	MT946222
14	TP11	2019.Apr.10	Beitun, Taichung, Taiwan	H9	III	MT946223
15	TP12	2019.Apr.03	Xihu, Miaoli, Taiwan	H9	III	MT946224
16	TP13	2019.Mar.28	Mingjian, Nantou, Taiwan	H9	III	MT946225
17	TP14	2019.Apr.09	Kaohsiung, Taiwan	H13	II	MT946226
18	TP15	2019.Apr.06	Shenzhen, China	H5		MT946227
19	TP16	2019.Apr.06	Shenzhen, China	H4		MT946228
20	TP17	NA	ChiangMai, Thailand	H1		MT946229
21	TP18	NA	ChiangMai, Thailand	H2		MT946230
22	TP19	2019.Apr.05	Puli, Nantou, Taiwan	H9	III	MT946231
23	TP20	2019.Apr.21	Jinsha, Kinmen, Taiwan	H7	IV	MT946232
24	TP21	2019.Apr.21	Jinsha, Kinmen, Taiwan	H11	I	MT946233
25	TP22	2019.Apr.21	Jinning, Kinmen, Taiwan	H13	II	MT946234
26	TP23	2019.May.14	Jhongpu, Chiayi, Taiwan	H13	II	MT946235
27	TP24	2019.May.13	Gongguan, Miaoli, Taiwan	H9	III	MT946236
28	TP25	2019.May.13	Xiangshan, Hsinchu, Taiwan	H11	I	MT946237
29	TP26	2019.May.13	Chaozhou, Pingtung, Taiwan	H13	II	MT946238
30	TP27	2019.May.19	Wenshan, Taipei, Taiwan	H9	III	MT946239
31	TP28	2019.May.17	Shilin, Taipei, Taiwan	H11	I	MT946240
32	TP29	2019.May.19	Wujie, Yilan, Taiwan	H9	III	MT946241
33	TP30	2019.May.27	Douliu, Yunlin, Taiwan	H9	III	MT946242
34	TP31	2019.May.23	Linnei, Yunlin, Taiwan	H9	III	MT946243
35	TP32	2019.Jun.05	Xiangshan, Hsinchu, Taiwan	H9	III	MT946244
36	TP33	2019.May.19	Minxiong, Chiayi, Taiwan	H13	II	MT946245
37	TP34	2019.Jun.09	Dahu, Miaoli, Taiwan	H9	III	MT946246

38	TP35	2019.May.19	Ji'an, Hualien, Taiwan	H11	I	MT946247
39	TP36	2019.May.22	Jinning, Kinmen, Taiwan	H10	I	MT946248
40	TP37	2019.May.29	Nanhua, Tainan, Taiwan	H9	III	MT946249
41	TP38	2019.May.16	Wufeng, Taichung, Taiwan	H13	II	MT946250
42	TP39	2019.May.31	Tianliao, Kaohsiung, Taiwan	H13	II	MT946251
43	TP40	2019.May.22	Jinsha, Kinmen, Taiwan	H10	I	MT946252
44	TP41	2019.Jun.24	Qiaotou, Kaohsiung, Taiwan	H13	II	MT946253
45	TP42	2019.Jul.26	Yanchao, Kaohsiung, Taiwan	H13	II	MT946254
46	TP43	2019.Jul.26	Neimen, Kaohsiung, Taiwan	H13	II	MT946255
47	TP44	2019.Aug.05	Jinning, Kinmen, Taiwan	H7	IV	MT946256
48	TP45	2019.Aug.05	Jinning, Kinmen, Taiwan	H7	IV	MT946257
49	TP46	2020.Jan.16	Tianliao, Kaohsiung, Taiwan	H13	II	MT946258
50	TP47	2020.Feb.23	Lucao, Chiayi, Taiwan	H9	III	MT946259
51	TP48	2020.Feb.29	Yuanli, Miaoli, Taiwan	H9	III	MT946260
52	TP49	2020.Mar.02	Wufeng, Taichung, Taiwan	H9	III	MT946261
53	TP50	2020.Mar.11	Neimen, Kaohsiung, Taiwan	H13	II	MT946262
54	TP51	2020.Mar.11	Xihu, Miaoli, Taiwan	H9	III	MT946263
55	TP52	2020.Mar.15	Beitun, Taichung, Taiwan	H9	III	MT946264
56	TP53	2020.Mar.16	Dahu, Miaoli, Taiwan	H9	III	MT946265
57	TP54	2020.Mar.17	Nanhua, Tainan, Taiwan	H13	II	MT946266
58	TP55	2020.Mar.20	Qishan, Kaohsiung, Taiwan	H13	II	MT946267
59	TP56	2020.Mar.21	Beitou, Taipei, Taiwan	H11	I	MT946268
60	TP57	2020.Mar.23	Shetou, Changhua, Taiwan	H9	III	MT946269
61	TP58	2020.Apr.05	Fanlu, Chiayi, Taiwan	H13	II	MT946270
62	TP59	2020.Mar.27	Linnei, Yunlin, Taiwan	H9	III	MT946271
63	TP60	2020.Mar.27	Yuanshan, Yilan, Taiwan	H3	V	MT946272
64	TP61	2020.Mar.29	Zuoying, Kaohsiung, Taiwan	H13	II	MT946273
65	TP62	2020.Apr.07	Ji'an, Hualien, Taiwan	H11	I	MT946274
66	TP63	2020.Apr.10	Tianliao, Kaohsiung, Taiwan	H13	II	MT946275
67	TP64	2020.Apr.10	Alian, Kaohsiung, Taiwan	H13	II	MT946276
68	TP65	2020.Apr.12	Fangliao, Pingtung, Taiwan	H13	II	MT946277
69	TP66	2020.Apr.13	Tamsui, New Taipei, Taiwan	H12	II	MT946278
70	TP67	2020.Apr.14	Wufeng, Taichung, Taiwan	H9	III	MT946279
71	TP71	2020.Apr.13	Neimen, Kaohsiung, Taiwan	H13	II	MT946280
72	TP73	2020.Apr.13	Jiali, Tainan, Taiwan	H13	II	MT946281
73	TP76	2020.Apr.13	Nanhua, Tainan, Taiwan	H13	II	MT946282
74	TP79	2020.Apr.14	Ren'ai, Keelung, Taiwan	H9	III	MT946283
75	TP81	2020.Apr.14	Xinyi, Keelung, Taiwan	H11	I	MT946284
76	TP82	2020.Apr.14	Xinyi, Keelung, Taiwan	H11	I	MT946285
77	TP83	2020.Apr.18	Nangang, Taipei, Taiwan	H11	I	MT946286
78	TP84	2020.Apr.18	Bali, New Taipei, Taiwan	H11	I	MT946287

79	TP85	2020.Apr.18	Bali, New Taipei, Taiwan	H9	III	MT946288
80	TP86	2020.Apr.18	Bali, New Taipei, Taiwan	H12	II	MT946289
81	TP87	2020.Apr.18	Bali, New Taipei, Taiwan	H11	I	MT946290

Table S2. Number of LSB eggs parasitized by "major" (*Anastatus fulloi* and *A. dexingensis*) and "minor" egg parasitoid species (*A. formosanus*, *A.* sp., *Ooencyrtus utetheisae*, *O. phongi* and eulophid wasp X). Note the egg numbers for the "minor" egg parasitoid species (in bold) are not included in the total egg numbers shown in Table 1.

		2018			2019		
		April	May	June	March	April	May
**Northern**							
	A.f.	40	40	1	0	0	39
	A.d.	13	264	369	0	0	28
	O.u.	0	**3**	0	0	0	0
	O.p.	0	0	0	0	0	0
	A. fo.	**1**	0	0	0	0	0
	A. sp.	0	0	0	0	**4**	0
	E.W.X	0	0	0	0	0	0
**Central**							
	A.f.	-	23	0	0	50	26
	A.d.	-	366	14	12	258	210
	O.u.	-	0	0	0	**5**	**7**
	O.p.	-	**5**	0	0	0	**5**
	A. fo.	-	0	0	0	0	0
	A. sp.	-	0	0	0	0	0
	E.W.X	-	**3**	0	0	0	0
**Southern**							
	A.f.	0	28	0	31	55	15
	A.d.	47	62	10	28	179	118
	O.u.	0	0	0	0	0	0
	O.p.	0	0	**5**	0	**1**	0
	A. fo.	0	0	0	0	**1**	0
	A. sp.	0	0	0	0	0	0
	E.W.X	0	0	0	0	0	0
**Kinmen**							
	A.f.	29	-	-	-	27	-
	A.d.	0	-	-	-	0	-
	O.u.	0	-	-	-	0	-
	O.p.	0	-	-	-	0	-
	A. fo.	0	-	-	-	0	-
	A. sp.	0	-	-	-	0	-
	E.W.X	0	-	-	-	0	-

A.f.: *Anastatus fulloi*; *A.d.*: *A. dexingensis*; *A.fo.*: *A. formosanus*; *A.* sp.: *Anastatus* sp.; *O.u.*: *Ooencyrtus utetheisae*; *O.p.*: *O. phongi*; *E.W.X*: eulophid wasp X
